# The Interplay of Perceptions and Conflict Behaviors during Disagreements: A Daily Study of Physical Teen Dating Violence Perpetration

**DOI:** 10.1177/08862605211021983

**Published:** 2021-06-02

**Authors:** Andréanne Fortin, Alison Paradis, Martine Hébert, Andréanne Lapierre

**Affiliations:** 1 Département de Psychologie, UQAM, Montréal, Québec, Canada; 2 Département de Sexologie, UQAM, Montréal, Québec, Canada

**Keywords:** Physical violence, dating relationships, conflict management, perceptions, adolescence

## Abstract

Physical dating violence (DV) is a widespread problem among adolescents. A growing body of literature demonstrates that physical DV often occurs during disagreements when partners use destructive conflict management strategies, such as conflict engagement (e.g., losing control, criticizing) or withdrawal (e.g., acting cold, being distant). However, little is known regarding how the individual daily variability on the use of destructive conflict management strategies can influence the probability of perpetrating day-to-day physical DV, especially if the other partner is also perceived as using destructive behaviors. Using an intensive longitudinal approach, the current study first aimed to examine the daily associations between the use of various conflict management strategies and physical DV perpetration in adolescent dating relationships. A second objective was to investigate if perceived partner’s conflict behaviors moderated the relation between self-reported conflict management strategies and day-to-day physical DV perpetration. A sample of 216 adolescents (*M_age_* = 17.03, *SD* = 1.49) involved in a dating relationship, completed a baseline assessment followed by 14 daily diaries. Results of multilevel logistic analyses revealed that using conflict engagement strategies significantly increased the probability of day-to-day physical DV perpetration. Furthermore, the probability of perpetrating physical DV was significantly higher on days in which teens reported using high levels of conflict engagement while also perceiving their partner as using high levels of conflict engagement or withdrawal. These findings yield new insights on the daily context in which disagreements might escalate into aggression. Evidence from this study further supports the conflict escalation pattern and the demand/withdraw communication pattern in the context of adolescent dating relationships. Preventive initiatives should address the interplay of perceptions and conflict behaviors concerning physical DV perpetration.

## Introduction

Early dating relationships not only offer opportunities for the development of identity and intimacy but also frequently become a central source of support and affection for adolescents (Furman et al., 2002). However, these relationships often come with their share of struggles and difficulties, especially regarding jealousy, trust, betrayal, or neglect (Fernet et al., 2016; McIsaac et al., 2008). Compared to romantic relationships in adulthood, teens report higher levels of conflictual interactions in their dating relationships (Lantagne & Furman, 2017). Difficulties in solving these interactions have been associated with the escalation of conflicts, which in return can lead to the occurrence of violent behaviors (De La Rue et al., 2017; Gonzalez-Mendez et al., 2018). Scholarly reports indicate that rates of dating violence (DV) are alarmingly high during adolescence (Taylor & Mumford, 2016; Wincentak et al., 2016). Conducted among a representative sample of adolescents from the province of Quebec, results from the Youths’ Romantic Relationships Survey indicated that 15% of adolescents reported being physically victimized by a dating partner in the past year (Hébert et al., 2017). As for perpetration, nationally available data revealed similar rates, with 18% of adolescents reporting having perpetrated physical DV towards a dating partner (Ybarra et al., 2016). Moreover, experiencing physical DV increases the likelihood of being involved in recurrent abusive relationships that may persist into adulthood (Cui et al., 2013; Taylor et al., 2017).

As physical DV often occurs during conflicts (Capaldi et al., 2012; Fernet et al., 2016), scholars have sought to gain a deeper understanding of conflict management processes among dating adolescents. Recent data suggests that adolescents who report perpetrating DV also describe using more destructive conflict management strategies (e.g., conflict engagement, withdrawal) over time than their non-perpetrator counterparts (Gonzalez-Mendez et al., 2018). However, no significant association for constructive conflict management strategies (e.g., compromise, collaboration) has been observed, suggesting that adolescents who report perpetrating DV do not differ from non-violent teenagers on this aspect (Gonzalez-Mendez et al., 2018). Furthermore, no significant differences were found regarding levels of care, love, and self-disclosure across violent and non-violent dating relationships (Giordano et al., 2010). These results notably suggest that positive dynamics are also observed among couples reporting the presence of DV, thus highlighting the complexity of this phenomenon. More studies are needed to improve our understanding of the dynamic processes associated with DV perpetration.

Conflict management depends on a variety of implicit factors and subjective cognitions, such as beliefs or expectations about conflict occurrence and its outcomes on the relationship, as well as perceived partner’s behaviors or intents (Feiring et al., 2018; Fernández-González et al., 2019; Verhoef et al., 2019). Attribution theory suggests that negative interpretations of another individual’s behaviors might influence their reactions towards them (Flynn & Graham, 2010). A systematic review on attributions and various aggressive behaviors in adulthood reported small to medium associations between negative attributions and aggression (Tuente et al., 2019). Thus, conflict behaviors adopted by a dating partner, if perceived as hostile, might exacerbate the probability of physical DV perpetration by entitling an aggressive response (Anderson & Bushman, 2002). Therefore, among adolescents’ daily conflicts and disagreements, it is likely that the use of destructive conflict management strategies will increase the probability of DV perpetration, especially if the partner is also perceived as using destructive behaviors. Surprisingly, this interplay between perceptions and conflict behaviors in relation to adolescent physical DV perpetration has yet been explored.

The high prevalence and the deleterious consequences associated with physical DV make it an important public health problem (Burczycka & Conroy, 2018; Butchart, & Mikton, 2014; Wincentak et al., 2016). However, despite significant advances in the field, much of the prior work remains cross-sectional, limiting our understanding of how DV arises over time. Dating partners who use severe forms of DV do not engage in violent behaviors every time a disagreement occurs in their relationship (Bartholomew et al., 2015; Finkel, 2007), suggesting that there is individual daily variability in the association between disagreements and DV. This hypothesis has yet been explored given that prior DV research has mostly relied on teen’s retrospective recall of events. Although retrospective self-report methods allow access to larger samples at a lower cost, they can be limited by memory biases, and more importantly, they preclude the exploration of the proximal factors associated with DV (Laurenceau & Bolger, 2005). In this regard, the use of daily diaries (i.e., several assessments in a short period of time) is gaining popularity in the field of social sciences research (Iida et al., 2012; Van Berkel et al., 2017). While reducing the time between the events of interest and collecting data, this method also makes it possible to examine individual’s behaviors, perceptions, and emotions in their daily context (Bolger et al., 2003; Laurenceau & Bolger, 2005). Therefore, using this innovative method could improve our understanding of micro-processes associated with conflict behaviors and DV perpetration during adolescence while considerably reducing the memory bias associated with the use of retrospective methods. Gaining a more in-depth understanding of why and when adolescents use DV is particularly relevant to inform prevention initiatives and promote healthy dating relationship behaviors among adolescents.

### The Current Study

Using an intensive longitudinal approach, this study aimed to examine associations between self-reported daily conflict management strategies and day-to-day physical DV perpetration. Since conflict management depends on a variety of implicit factors and subjective perceptions, this study also aimed to investigate how perceived partner’s behaviors during daily disagreements moderate the relation between self-reported use of conflict management strategies and physical DV perpetration. By examining the interplay between one’s own behaviors and perceptions, the current study will contribute to a more dynamic investigation of the context in which conflicts escalate into violence during adolescence.

## Method

### Participants

The sample consisted of 216 adolescents (range = 14–19 years, *M*_age_ = 17.03 years, *SD* = 1.49) who completed 14 daily diaries, including 92 boys (42.6%), most of whom had parents of Canadian origin (71.9%) and spoke French at home (85.3%). To participate, adolescents had to be currently involved in a dating relationship for at least one month without cohabiting with their partner. A significant proportion of adolescents reported being in that relationship for more than a year (41.2%). Statistical power for our multilevel models was estimated based on a previous simulation study (Arend & Schäfer, 2019). Based on their results, to achieve 80% power at 0.05, with a medium-sized intraclass correlation coefficient (ICC = 0.38), 2.5 Level-1 observations (i.e., mean number of disagreements per participant) and 173 Level-2 participants, our model can detect effect sizes starting from *r* = 0.14 for Level-1 direct and interactive effects and *r* = 0.28 for Level-2 direct effect, which are in the small to medium range.

### Procedure

Adolescents were recruited on a voluntary basis in high schools and colleges from the Montreal area, and through social networks (i.e., Facebook). When adolescents were recruited in educational institutions, research assistants either presented the project in classrooms or at an information booth in the schools’ common areas (e.g., cafeteria). The research procedure was presented to interested participants and written consent was obtained. When recruited online, youth were asked to leave their contact information, so research assistants could reach them and go through the same procedure with them over the phone. After consenting, adolescents were invited to fill out an initial online survey (30–45 min.). Then, for 14 consecutive days, they completed daily diaries (5 min.) sent via short message service (SMS) or e-mail. Teens were asked to complete the diaries between 8:00 pm (i.e., when they received the questionnaire) and 9:00 am the next day. Follow-up phone calls were made by research assistants with each participant on the 2nd, 7th, and 12th days of the study to promote participants’ retention and ensure that their participation was going well. Participants received $4 for each questionnaire they completed, for a maximal amount of $60 (initial baseline survey and 14 daily diaries). Participants who completed at least 12 out of 14 daily diaries were also eligible to win a $250 gift certificate for the mall of their choice. The institution’s human research ethics committee of the Université du Québec à Montréal granted ethical approval of the project.

## Measures

### Sociodemographic data.

Sociodemographic information was collected during the baseline assessment. Adolescents’ age, sex at birth and length of their current relationship were used as control variables in all subsequent analyses.

### Daily conflict management strategies.

Every day, adolescents indicated if they had any disagreements with their dating partner using a single item (dichotomous yes/no response). When participants answered “yes”, they were then asked to report on the strategies used by both partners to solve the most important disagreement they had on that day. Then, a French adaptation of the Conflict Resolution Styles Inventory (Fortin et al., 2020; Kurdek, 1994) measured each of the three strategies used during that disagreement: *Conflict engagement* (four items, e.g., launching personal attacks), *Withdrawal* (four items, e.g., tuning the other person out) and *Problem-solving* (eight items, e.g., trying to find the right balance between the two positions). Adolescents were asked to answer each of the 16 items twice, first in relation to their conflict behaviors toward their dating partner and second in relation to their perception of their partner’s conflict behaviors toward them. In order to limit the response burden for the adolescents, a 3-point rather than a 5-point Likert scale was used in the daily diaries. Response choices ranged from 1 (not at all) to 3 (very much). Internal consistency was adequate with Omega coefficients (*ω*; McDonald, 1999) ranging from 0.75 to 0.87 at the within-level (i.e., items’ response consistency by the same individual over the 14 days) and from 0.90 to 0.97 at the between-level (i.e., items’ response consistency across the entire sample).

### Daily physical DV perpetration.

Daily physical perpetration of DV was measured using four items from the physical assault subscale of the widely known Revised Conflict Tactics Scales (CTS2; Lussier, 1998; Straus et al., 1996). This scale has been used extensively in the past with various populations, including adolescents (e.g., Cascardi et al., 1999; Muñoz-Rivas et al., 2007; Nocentini et al., 2011). In order to obtain a brief daily measure of physical DV perpetration, four items (i.e., “Forcefully pushing or shoving,” “Slapping or hitting,” “Throwing items that could hurt,” and “Kicking, biting, or hair-pulling”) were selected from the physical assault subscale of the original instrument (Straus et al., 1996). These items have been previously used to successfully assess physical DV (Simon & Furman, 2010). On days in which in-person disagreements occurred, participants were asked to indicate how often they had engaged in the various violent behaviors during the disagreement. Again, a 3-point Likert scale was preferred to mirror the strategy used with the CRSI. Response choices ranged from 1 (never) to 3 (three times or more). For the present analyses, a dichotomous score was created, indicating whether participants endorsed perpetrating any of the items on that particular day. Dichotomous scores on the daily victimization subscale were used as a control variable in subsequent analyses.

#### Statistical Analysis

Preliminary analyses were conducted using SPSS 27. Due to the data’s hierarchical structure (i.e., days nested within individuals), a two-level model with random intercept was estimated using Mplus 8.3 (Muthén & Muthén, 2017). An essential step in intensive longitudinal analyses is to divide the data into between-person (i.e., variability in average scores between participants or inter-person differences) and within-person components (i.e., variability in each participant’s daily scores or intra-person differences) to analyze the Level-1 predictors (McNeish & Hamaker, 2020). In the current study, a two-level model with fixed and random effects (i.e., residuals) was conducted to simultaneously examine the within- and between-person main effects of self-reported conflict management strategies (i.e., conflict engagement, withdrawal and problem-solving) on physical DV perpetration. Moderation analysis with partner-perceived conflict behaviors (i.e., conflict engagement, withdrawal and problem-solving) was then conducted on the significant associations observed between conflict management strategies and physical DV perpetration.

To examine the between-person effects, self-reported conflict management strategies (Level-1 variables) were grand-mean centered by subtracting the sample’s grand mean (i.e., all measurement points across all individuals in the sample) from the average score of each participant on the Level-1 predictor over the 14 days. To examine the within-person effects, self-reported and partner-perceived conflict management strategies were person-mean centered, meaning that each participant’s average value on the Level-1 predictor and moderator across all measurement points was subtracted from their raw value at each measurement point.

All multilevel models were estimated using maximum likelihood estimator with robust standard errors (i.e., MLR) to account for non-normality of data (Lanza & Cooper, 2016) and a logit link to examine DV perpetration as a dichotomous outcome. Since the outcome variable is dichotomous at Level-1 but is considered continuous at Level-2 (i.e., aggregation of the total number of days of physical DV perpetration within days of conflict), odds ratios were calculated for the within-person main effects and for the interaction effects only (Level-1). Analyses were conducted on days in which adolescents reported having an in-person disagreement with their partner (*n* = 434). Missing data from these 434 observations were handled using full information maximum likelihood (FIML; Muthén & Muthén, 2017). Covariates were added in all multilevel models to control for the fixed and random effects of time and daily physical DV victimization at the within-person level, and the fixed effects of sex at birth, age, and relationship length at the between-person level. Covariates were trimmed from the final models if not significant to avoid overcontrolling the model (Little, 2013). Model equations for the interaction models and the syntax used to estimate all multilevel models are provided in the Supplemental Material (available online).

## Results

### Descriptive Statistics

Compliance with daily reports was excellent and reached 92.4% across the 14 days as adolescents reported on 2,795 days out of a possible 3,024 entries (range = 2–14, *M* = 12.94 daily diaries, *SD* = 2.27). Teens reported disagreements on average 2.73 days over the 14 days (*n* = 590 days). Adolescents reported using problem-solving strategies in almost every disagreement (97.1%), withdrawal strategies in 58.1% and conflictual behaviors in 34.1% of them. Rates for perceived partner behaviors were similar. Perpetration of DV was reported by 9.2% of participants (*n* = 18) for a total of 22 days over the 14-days period. Similar rates were reported for physical victimization (8.7%), corresponding to 17 adolescents over 21 days. Table 1 presents the mean, standard deviation, and between-person correlations of each study variable.

**Table 1.** Descriptive Statistics and Between-Person Correlations Among Study Variables.

Variables	Descriptive		Correlations					
	Mean	*SD*	1	2	3	4	5	6
Self-reported								
Conflict engagement	1.21	0.39	–					
Withdrawal	1.45	0.54	0.41**	–				
Problem-solving	2.25	0.55	–0.14**	–0.39**	–			
Physical DV perpetration	0.05	0.22	0.35**	0.15**	–0.13**			
Partner-perceived								
Conflict engagement	1.22	0.39	0.60**	0.37**	–0.09*	0.24**	–	
Withdrawal	1.38	0.50	0.32**	0.40**	–0.15**	0.39**	0.11*	–
Problem-solving	2.14	0.59	–0.13**	–0.33**	0.77*	–0.27**	–0.36**	–0.13**

*Note*. ***p < .001, **p < .01, *p < .05; Conflict management scales ranged from 1 to 3; Point-biserial correlations were computed for physical DV perpetration (dichotomous variable).

### Conflict Behaviors on Physical DV Perpetration

Table 2 presents estimates of fixed and random effects of self-reported conflict management strategies on daily physical DV perpetration. Multilevel analyses indicate a main effect of self-reported conflict engagement strategies on physical DV perpetration at the within- and between-person level. Results at the within-person level showed that conflict engagement is associated with physical DV perpetration at the daily level (*b* **=** 1.10, *SE* **=** 0.49, *p* **=** .025). This indicates that on days in which adolescents reported a one unit increase on their self-reported use of conflict engagement (i.e., compared to their own average over the 14 days), the daily odds of perpetrating physical DV during the same event significantly increased by 3 times. Results at the between-person level indicate that adolescents who reported using higher levels of conflictual strategies compared to the sample’s grand mean also reported more frequent physical DV perpetration during conflictual interactions across the 14 days (*b* **=** 5.83, *SE* **=** 1.74, *p* **=** .001). On the other hand, no significant main effects were found regarding self-reported use of withdrawal or problem-solving conflict management strategies at the within- or between-person level.

**Table 2.** Main Effects of Self-Reported Conflict Management Strategies on Daily Physical DV Perpetration.

Parameters	β	*b* (*SE)*	95% CI	*p*	*R^2^*
Intercept	1.73	3.78 (0.94)	[1.93, 5.62]	0.000	
*Within-person*					17.9%
Conflict engagement	0.15	**1.10 (0.49)**	[0.14, 2.05]	**0.025**	
Withdrawal	0.23	1.26 (0.74)	[–0.19, 2.70]	0.089	
Problem-solving	–0.19	–1.19 (1.04)	[–3.24, 0.85]	0.253	
*Between-person*					74.7%
Conflict engagement	0.76	**5.83 (1.74)**	[2.42, 9.24]	**0.001**	
Withdrawal	–0.31	–1.61 (1.19)	[–3.95, 0.73]	0.178	
Problem-solving	–0.29	–1.35 (0.88)	[–3.07, 0.37]	0.125	
Residual	0.25	1.21 (1.93)	[–2.58, 4.99]	0.532	

*Note*. Length of the relationship was the only significant covariate; nonsignificant covariates were removed from the final model.

### Moderating Role of Perceived Partner’s Conflict Behaviors

Since conflict engagement was the only self-reported strategy significantly associated with physical DV perpetration, moderation analyses were then conducted to examine the interactive effect of each perceived partner’s conflict behaviors (i.e., conflict engagement, withdrawal, and problem-solving) on the relation between self-reported conflict engagement and daily physical DV perpetration. Three independent models including one interaction term were estimated.

Results of the interactions between self-reported conflict engagement and partner-perceived conflict behaviors on daily physical DV perpetration are presented in Table 3. Results suggest that the probability of perpetrating physical DV was significantly higher on days in which adolescents used high levels of conflict engagement while also perceiving high levels of conflict engagement from their partner. More specifically, this indicated that the interplay between both partner’s conflictual behaviors increases the probability of day-to-day physical DV perpetration by 1.65 times (*b* **=** 3.52, *SE* **=** 1.40, *p* **=** .01). Significant results for the interaction with perceived partner’s withdrawal were also observed. Results indicate that the likelihood of daily physical DV perpetration increases by 1.29 times when adolescents reported using high levels of conflict engagement while also perceiving their partner as using high levels of withdrawal strategies (*b* **=** 3.20, *SE* **=** 1.62, *p* **=** .049). No significant interaction was found between self-reported conflict engagement and partner-perceived problem solving (*b* **=** –1.52, *SE* **=** 1.75, *p* **=** .384).

**Table 3.** Interactive Effects of Self-Reported Conflict Engagement and Partner-Perceived Conflict Management Strategies on Daily Physical DV Perpetration.

**Parameters**	β	*b* (*SE)*	95% CI	*p*	*R^2^*
*Model 1. Interaction with P conflict engagement*					
Intercept	3.07	**5.34 (1.05)**	[3.28, 7.40]	**0.000**	
*Within-person*					8.6%
S conflict engagement	0.07	0.47 (0.78)	[–1.06, 2.01]	0.547	
P conflict engagement	0.12	0.98 (1.21)	[–1.39, 3.36]	0.417	
S × P	0.21	**3.52 (1.40)**	[0.86, 6.18]	**0.010**	
*Between-person*					78.8%
S conflict engagement	0.71	**4.27 (1.12)**	[2.07, 6.47]	**0.000**	
Residual	0.21	0.64 (1.58)	[–2.45, 3.73]	0.684	
*Model 2.Interaction with P withdrawal*					
Intercept	5.53	**11.58 (3.10)**	[5.51, 17.66]	**0.000**	
*Within-person*					13.5%
S conflict engagement	0.11	0.76 (0.61)	[–0.42, 1.95]	0.207	
P withdrawal	0.22	1.17 (0.58)	[0.02, 2.31]	0.046	
S × P	0.22	3.20 (1.62)	[0.02, 6.38]	0.049	
*Between-person*					63.4%
S conflict engagement	0.68	**4.94 (1.48)**	[2.03, 7.85]	**0.001**	
Residual	0.37	1.60 (2.80)	[–3.88, 7.09]	0.566	
*Model 3.Interaction with P problem-solving*					
Intercept	4.45	**10.03 (2.58)**	[4.97, 15.08]	**0.000**	
*Within-person*					12.9%
S conflict engagement	0.21	**1.54 (0.68)**	[0.21, 2.88]	**0.024**	
P *problem-solving*	–0.25	–1.36 (0.77)	[–2.87, 0.16]	0.080	
S × P	–0.09	–1.52 (1.75)	[–4.96, 1.91]	0.384	
*Between-person*					60.0%
S conflict engagement	0.66	**5.24 (1.51)**	[2.29, 8.20]	**0.001**	
Residual	0.40	2.03 (2.65)	[–3.16, 7.22]	0.442	

*Note.* S = Self-reported, P = Partner-perceived; Length of the relationship was the only significant covariate, nonsignificant covariates were removed from the final models.

**Figure 1.**
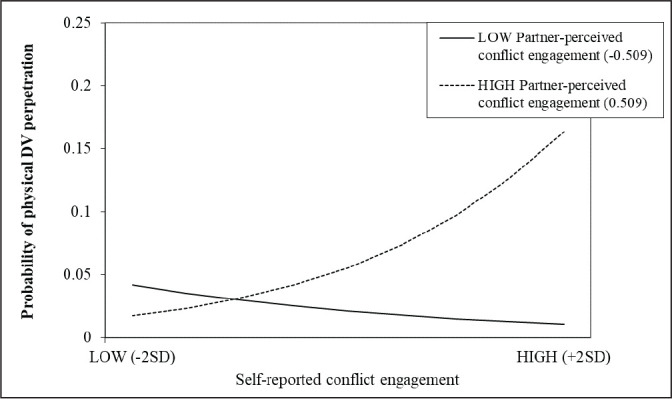
Simple Slopes of the Interaction Between Self-Reported and Partner-Perceived Conflict Engagement on the Probability of Physical DV Perpetration.

**Figure 2.**
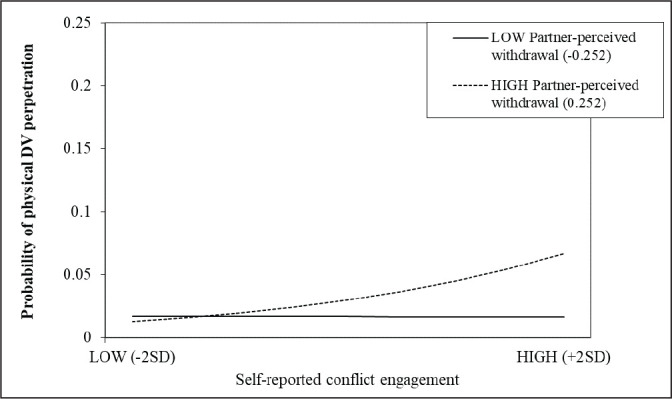
Simple Slopes of the Interaction Between Self-Reported Conflict Engagement and Partner-Perceived Withdrawal on the Probability of Physical DV Perpetration.

The Johnson-Neyman technique (Johnson & Neyman, 1936; Preacher et al., 2006) was used to probe the interaction between self-reported partner-perceived conflict behaviors on the daily probability of physical DV perpetration. More specifically, this technique, which uses 95% confidence bands around the simple slope to determine the region of significance across the full range of conditional values of the moderator, was used to estimate the specific value of the moderator at which the simple slope is significantly different from 0 (i.e., at which the interactive effect becomes significant). Graphs showing the region of significance for each interaction are provided as Supplemental Material. The specific value of the moderator was then used to plot the simple slopes for high and low levels (+/–2 *SD*) of self-reported partner-perceived conflict behaviors on the daily probability of physical DV perpetration. The slope of physical DV perpetration on self-reported conflict engagement becomes significant for levels of partner-perceived conflict engagement higher than 0.509 units above the individual mean across the 14 days (Figure 1; slope of high partner-perceived conflict engagement). Whereas for partner-perceived withdrawal, the interaction is significant when levels of the moderator are higher than 0.252 units above the individual mean across the 14 days (Figure 2; slope of high partner-perceived withdrawal). Meaning that the probability of perpetrating DV significantly increases on days in which adolescents reported using higher levels of conflict engagement than their usual mean across the 14 days while also perceiving their partner as using higher levels of conflict engagement (i.e., 0.509 unit higher) or withdrawal (i.e., 0.252 unit higher) strategies than they usually do.

## Discussion

This study aimed to contribute to a more dynamic investigation on the contexts in which daily disagreements escalate into physical violence during adolescence. More specifically, this study examined the distinct contribution of self-reported conflict engagement, withdrawal, and problem-solving strategies as well as perceived partner conflict behaviors in association with daily physical DV perpetration. Results of this study add depth to existing knowledge on the link between destructive conflict management strategies and violence in adolescence as it is, to our knowledge, the first investigation to examine how conflict behaviors relate to physical abuse in adolescent dating relationships using an intensive longitudinal approach.

Daily reports indicated that disagreements with a dating partner occurred on 19.5% of days (*n* = 590), with 434 days characterized by in-person disagreement. Among these 434 disagreements, 5.1% resulted in physical DV perpetration, with 9.2% of adolescents reporting the use of physical DV at least once across the 14 days. On the one hand, compared with the lifetime prevalence of physical DV perpetration, the rate observed across the two weeks of daily diaries is particularly high considering it represents a mere snapshot of adolescents’ daily lives. On the other hand, this is consistent with research on the assessment of DV indicating that violent acts are often underestimated (Waterman et al., 2019). Because the daily diaries method allows behaviors that might go otherwise underreported to be captured, the higher rates observed using this method likely provide a more accurate estimate of DV than retrospective surveys (Krenek et al., 2016). This is alarming and highlights the need to develop early effective prevention initiatives to be implemented starting in adolescence.

Consistent with study hypotheses, we found that self-reported use of conflict engagement significantly heightened the likelihood of adolescents perpetrating physical DV over the 14 days. Overall, this suggests that adolescents who use higher levels of conflict engagement than their peers have an increased risk of perpetrating physical DV. Furthermore, the within-person association of conflict engagement and physical DV suggests that on days in which adolescents increased their own level of conflict engagement, the probability of resorting to violent behaviors also rises. As such, in addition to whether adolescents generally use high or low levels of conflict engagement, their daily variations in conflict management seem to be of great interest in understanding the context in which teen DV occurs. This suggests that even adolescents who do not frequently resort to conflict engagement strategies could be at risk of perpetrating physical DV on days they use more conflict engagement than what they usually do.

Results from the interactions further suggest an interrelation between both partner’s use of conflict engagement strategies. Evidence from the current study revealed that the probability of perpetrating physical DV was significantly higher when adolescents used conflict engagement while also perceiving their partner as using conflictual behaviors. This result gives us a powerful insight into the daily context in which conflict escalation might lead to aggression. That is, perceiving both oneself and one’s partner as actively and negatively engaging in an argument significantly increases the probability of conflict escalating into physical DV, thus suggesting a pattern of conflict escalation. This is consistent with results from prior cross-sectional research, which found an association between the reciprocal use of conflict engagement and the occurrence of abuse in adult couples (Bonache et al., 2019).

Contrary to expectations, no significant associations were found regarding one’s own use of withdrawal strategies, therefore suggesting that trying to avoid daily disagreements may not be associated with physical DV perpetration. This is somewhat surprising considering that previous studies showed that the withdrawal subscale of the Conflict Resolution Styles Inventory (Kurdek, 1994) was useful in distinguishing between adolescents involved in a violent relationship (Bonache et al., 2016). There are several possible explanations for this result. In the study conducted by Bonache et al. (2016), conflict management strategies and DV were assessed over the past 12 months, rather than over the past 24 hours. Given such a long reference period, it is possible that withdrawal and physical DV behaviors did not occur simultaneously. Therefore, Bonache et al.’s (2016) results might reflect a more distal communication pattern in which DV perpetration is used as a last resort to preserve one’s intimacy against a pressuring partner. Moreover, in their study, both psychological and physical DV were assessed and used as one global measure of DV, whereas only physical DV was examined in the current study. As such, withdrawal behaviors could be significantly associated with the use of psychological rather than physical DV.

On the other hand, results from the current study showed an interaction between self-reported conflict engagement and perceived partner’s withdrawal, suggesting that the probability of physical DV perpetration was significantly higher on days in which adolescents reported engaging negatively in a disagreement while perceiving their partner as shutting down from the argument. Hence suggesting the presence of a demand/withdraw communication pattern in which one partner criticizes and pressures the other to engage in the disagreement while the other partner withdraws, stays silent and defensive, or refuses to further discuss (Christensen & Heavey, 1990). This pattern has been widely documented in adult intimate relationships (Holley et al., 2010; Schrodt et al., 2014) and has been associated with psychological distress and the occurrence of violent behaviors (Eldridge et al., 2007; Fournier et al., 2011). Consistent with prior research, evidence from the current study supports the existence of a demand/withdraw communication pattern among adolescent dating relationships. As few studies have examined this pattern in dating relationships, future research is needed to replicate this finding.

Adolescents from our sample indicated using constructive conflict management strategies in almost every disagreement they reported. This is consistent with prior findings suggesting that in order to preserve their relationships, adolescents tend to use more constructive strategies to manage disagreements with their dating partner than with their friends (Laursen et al., 2001). Problem-solving strategies were expected to reduce the daily probability of physical DV perpetration, but no significant associations were observed. Perceived partner’s constructive strategies were also examined as moderators between self-reported conflict engagement and physical DV perpetration. Again, no significant effects were observed. The lack of variability in the sample’s score of problem-solving strategies may explain the absence of significant association with physical DV perpetration. This is consistent with previous work on DV in which adolescents who reported perpetrating DV did not differ from their non-violent counterparts regarding the use of constructive conflict management strategies (Gonzalez-Mendez et al., 2018). The present findings notably suggest that regardless of whether or not adolescents use constructive strategies to manage disagreements with their dating partner, using destructive conflict behaviors is the crucial element that significantly increases their chances of perpetrating DV (Gonzalez-Mendez et al., 2018; Smith-Darden et al., 2017). Moreover, the fact that couples disclosing the presence of DV also report using constructive strategies to manage conflicts, as well as high levels of care, love, and self-disclosure in their relationship (Giordano et al., 2010) might make the decisional process of ending an abusive relationship hard for adolescents. Current results do not support the idea that constructive strategies serve as a protective factor for daily physical DV perpetration, yet they highlight the complexity of this phenomenon during adolescence.

By providing evidence from an adolescent sample, the current findings enhance our comprehension of conflict management and DV perpetration within an understudied population who have been more difficult to engage in research projects. Results are largely consistent with empirical reports with adult and young adult samples (Bonache et al., 2019) and support the presence of various communication patterns in adolescents couples, namely the conflict escalation and the demand/withdraw patterns. The diverse constitution of the sample that included high ratio of boys (40%) and ethnocultural diversity (i.e., 30% reporting that their parents had an ethnocultural affiliation other than Canadian) also has implications for diversity as it allows for a greater generalization of the results among adolescent dating relationships.

Still, these conclusions should be considered in light of some limitations. As this study aimed to examine the role of perceptions regarding physical DV perpetration, the respondent’s perceptions were not matched with the actual behaviors adopted by their partner. Therefore, using dyadic reports in future research would be interesting to compare the effect of one’s perception versus the behaviors reported by the other partner. Dyadic reports may also provide more accurate data on the behaviors adopted by both partners and offer meaningful information on the conflict management process at play among adolescent couples. Another important limitation concerns the assessment of DV. In the current study, only physical DV perpetration was assessed within in-person disagreements. Even if DV often occurs during conflicts (Capaldi et al., 2012), other forms of DV, such as sexual and psychological DV, might happen in other contexts which were not considered in the present study. Sexual and psychological DV are recognized as quite common in first dating relationships (Hébert et al., 2017, 2019). As such, it would be useful for future studies to examine a broader array of contexts and violent behaviors. Further, because of the low frequency of in-person conflicts, the within-person level included only a small number of observations (i.e., a mean of 2.5 observations per participant). Power analysis revealed that the models were adequately powered, but considering the rare frequency of daily physical DV perpetration (i.e., 22 violent events out of 434 in-person conflicts), future studies assessing adolescent samples over a longer period of time are needed to replicate the current findings.

Moreover, in order to promote the development of healthy relationships from adolescence, focus also needs to be directed on protective factors. As the current results did not support the hypothesis that constructive strategies may act as a buffer between self-reported conflict engagement and physical DV perpetration, future studies should aim at identifying daily protective factors. Finally, one major limitation of the current study is that sex differences were not examined. Based on results from previous studies (Bonache et al., 2017), it is possible to hypothesize that a three-way interaction between sex, self-reported, and perceived-partner destructive conflict management strategies on physical DV perpetration would have yielded significant results. Yet, the sample size was not sufficient to test such an interaction since it has been demonstrated that the number of observations required for testing a three-way interaction has to be four times as large as what is required for testing a two-way interaction (Heo et al., 2014). Further studies are clearly needed to better understand the differences between boys and girls during daily disagreements and should include adolescents from sexually diverse backgrounds.

## Implications and Conclusion

Gaining an in-depth understanding of why and when adolescents adopt violent behaviors undoubtedly helps to prevent conflict escalation into DV. Since within-individual variations in the use of conflictual strategies were associated with physical DV, meaning that using higher levels of conflict engagement than one’s own average increases the probability of perpetrating physical DV, results from the present study support the need for universal DV prevention initiatives in which all adolescents are targeted for intervention. Evidence from the present study also highlights that both conflict behaviors and perceptions are associated with daily perpetration of physical DV during adolescence. By increasing adolescent’s awareness of their own behaviors and perceptions during daily interactions with their dating partner, this new knowledge can help adolescents recognize situations in which they are at higher risk of using physical DV. Findings from the present study yield new insights on the daily context in which conflicts escalate into DV by improving our understanding of the dynamic processes associated with DV perpetration. As such, using these findings to inform future prevention initiatives will provide means to sustain positive and healthy dating relationships in adolescents.

## Author Biographies

**Andréanne Fortin, B.Sc.,** is a PhD student in the Department of Psychology at Université du Québec à Montréal. Her research focuses on the context in which daily conflicts escalate into physical dating violence during adolescence. She also examines the interplay between both partners’ strategies used during conflicts.

**Alison Paradis, PhD,** is an associate professor in the Department of Psychology at Université du Québec à Montréal. Her main research interest focuses on interpersonal violence, including childhood violence exposure and intimate partner violence. She is currently interested in the etiology and prevention of dating violence in adolescence and young adulthood.

**Martine Hébert, PhD,** is a full professor in the Department of Sexology at Université du Québec à Montréal. She holds the Tier I Canada Research Chair on Interpersonal Trauma and Resilience and leads the FRQ-SC sexual violence and health team. Her main research interests relate to child sexual abuse and adolescent dating violence.

**Andréanne Lapierre, B.Ed.,** is a PhD candidate in the Department of Psychology at Université du Québec à Montréal. Her research focuses on the impact of daily variations of stress and romantic attachment levels on teen dating violence.
